# A Service-Learning Program Based on Comprehensive Geriatric Assessment and Health Promotion in Older Adults: Protocol for the GEROS Project

**DOI:** 10.1186/s12877-026-07312-1

**Published:** 2026-03-21

**Authors:** Júlia Casacuberta-Roca, Eduard Minobes-Molina, Marc Terradas-Monllor, Anna Escribà-Salvans, Vinicius Rosa Oliveira, Elisenda Fenollosa-Romaní, Meritxell Mondejar-Pont, Emília Chirveches-Pérez, Núria Gorchs-Font, Ester Goutan-Roura, Marc Vila, Sergi Blancafort-Alias, Marta Dachs-Delgado, Patricia Lizett Vilca Salazar, Judit Rusiñol-Rodríguez, Alicia García-Álvarez, Tony Valente, Marta Casals-Coll, Clara Madrid-Alejos, Javier Jerez-Roig

**Affiliations:** 1 Research group on Methodology, Methods, Models and Outcomes of Health and Social Sciences (M₃O), Faculty of Health Sciences and Welfare, Institute for Research and Innovation in Life Sciences and Health in Central Catalonia (IRIS-CC), University of Vic-Central University of Catalonia (UVic-UCC), Vic, Spain; 2 Research group on Methodology, Methods, Models and Outcomes of Health and Social Sciences (M₃O), Faculty of Health Sciences and Welfare. Centre for Health and Social Care Research (CESS), University of Vic-Central University of Catalonia (UVic-UCC), Vic, Spain; 3 Research Group on Attention to Diversity (GRAD), Faculty of Education, Translation, Sports and Psychology, Institute for Research and Innovation in Life Sciences and Health in Central Catalonia (IRIS-CC), University of Vic- Central University of Catalonia (UVic-UCC), Vic, Spain; 4 Faculty of Education, Translation, Sports and Psychology, University of Vic- Central University of Catalonia (UVic-UCC), Vic, Spain; 5 Catalan Institute of Oncology (ICO), l’Hospitalet de Llobregat, Barcelona, Spain; 6 Equip d’Assistència Primària Vic (EAPVIC), Institute for Research and Innovation in Life Sciences and Health in Central Catalonia (IRIS-CC), Vic, Spain; 7 Institute of Sport Science and Innovations, Lithuanian Sports University, Kaunas, Lithuania

**Keywords:** Ageing, Comprehensive geriatric assessment, Education, Frailty, Health sciences, Service-learning, Undergraduate health students

## Abstract

**Background:**

Population ageing presents increasing challenges for health systems. Identifying complex needs and planning individualized care through Comprehensive Geriatric Assessment (CGA), promoting intergenerational engagement and empowerment via Service-Learning, are key strategies to support healthy and active aging. The general aim of the study is to evaluate the impact of a Service-Learning program that brings undergraduate health students into contact with community-dwelling older adults, assessing its benefits for students’ professional development and for older adults’ knowledge regarding their health.

**Methods:**

Research protocol for Service-Learning program with a mixed methodology and experimental intervention design, involving undergraduate students from Nursing, Physiotherapy, Human Nutrition and Dietetics, Occupational Therapy and Psychology degrees, and older adults from the community over 60 years old. The intervention at the university, consisting of three sessions, will be based on CGA. Student data will be collected during the final session using the questionnaire developed and validated by León-Carrascosa et al. (2020) to evaluate Service-Learning in higher education. After the first and last session, an additional questionnaire will assess satisfaction and perceived competency development. Older participants will be assessed with VIG-Frail, Integrated Care of Older People (ICOPE) screening tool, EuroQol 5D-5L, Nursing Outcomes Classification and sociodemographic data. They will complete a post intervention questionnaire regarding their satisfaction, perceived health status, knowledge of healthy lifestyle habits, and awareness of community resources, whose items will be evaluated using a 5-point Likert scale. Data will be collected through the REDCap platform and analysed using SPSS. Qualitative data from focus groups will undergo thematic analysis.

**Discussion:**

This study seeks to enhance both education and community health by integrating Service-Learning into health sciences training aligned with the WHO ICOPE framework. It aims to assess students’ perceptions of their preparation in geriatric assessment and person-centred care, while supporting early frailty detection and promoting healthy ageing in the community. Overall, it contributes to preparing future professionals for population ageing.

**Trial registration:**

This study was registered in *Clinical Trials* with the registration number NCT06890325 on March 24, 2025. As the registration occurred after the enrolment of the first participant, the trial was retrospectively registered.

**Supplementary Information:**

The online version contains supplementary material available at 10.1186/s12877-026-07312-1.

## Background

Population aging is a current global challenge, driven by increased life expectancy and declining fertility rates. Between 2015 and 2050, the percentage of the world’s population over the age of 60 will nearly double [1], with a direct impact on healthcare systems. In Catalonia (Spain), a similar trend is observed, with a rising dependency rate among older adults, posing a challenge for healthcare and social policies [2–6]. In Osona, Central Catalonia region, the population tends to increase, with the most significant growth observed among older age groups, particularly those aged 65 and over, who represented 18.6% of the population in 2024. Projections suggest that this trend of population ageing will continue in upcoming years [7, 8]. In this context, a progressive decline in physical and cognitive functions of older adults will lead to a greater population risk of frailty. In the first decade of the 2000s, the concept of frailty began to emerge to describe individuals undergoing the aging process. Frailty is defined as the geriatric syndrome characterized by a reduced physiological capacity to respond to external stressors, which increases the individual’s risk of falls, functional decline, disability, dependence and institutionalization [9]. Its prevalence ranges between 10 and 15% among community individuals aged 65 and over in Europe [10]. It has been described that the early detection of frailty enables a comprehensive approach, allowing individuals to maintain autonomy and improve quality of life for as long as possible [11]. Frailty often precedes dependency, when older individuals require assistance with daily activities. In more advanced stages, disability may develop, limiting individuals’ ability to live independently and affecting their quality of life.

These age-related conditions place significant pressure on healthcare and social care systems. Hospitals and long-term care facilities are increasingly required to provide complex, continuous care, while social services must respond to the growing demand for home support, community-based programs, and caregiver assistance. Furthermore, the rising costs associated with managing chronic diseases and age-related decline highlight the urgent need for preventive strategies, integrated care models and policies that promote healthy ageing. This situation points out the need for a shift from a traditional model of care, focused primarily on health problems, to one centred on the preservation of functional ability defined as the health-related attributes that enable individuals to be and act with that they value [12]. To develop person-centred intervention plans, it is essential to conduct assessments that enable the early detection of frailty and the design of preventive strategies to promote healthy ageing. Comprehensive Geriatric Assessment (CGA) is the gold standard of evaluating older adults, serving as a multidisciplinary diagnostic process that assesses medical, functional, psychological, and social capacities to eventually determine frailty status and various geriatric syndromes [13]. The use of CGA as a preventive tool in community older adults has been explored for several decades. Early studies already demonstrated the potential of CGA-based interventions to preserve functional capacity and delay disability [14]. Building on this foundation, the World Health Organization (WHO) established in 2015 a framework for action on ageing and health that set a goal of promoting and maintaining individuals. Functional ability is composed of intrinsic capacity, the environment, and the interaction between the individual and their environment. Intrinsic capacity refers to the composite of all the physical and mental capacities of an individual and represents the set of resources a person can draw on throughout life. The goal of intrinsic capacity is to measure individual’s capacities, not their deficits [15].

These new concepts of functional ability and intrinsic capacity call for their promotion and optimization as a strategy to prevent or slow the decline of these capacities over time. In this line, the WHO, in 2017, published a multidisciplinary guideline for the Integrated Care for Older People (ICOPE), which includes five key domains to evaluate the intrinsic capacity: cognition (cognitive decline), locomotor capacity (limited mobility), vitality (undernutrition), vision (vision impairment), hearing (hearing loss) and psychological capacity (depressive symptoms). Although the ICOPE screening tool is not yet validated, it is gaining increasing relevance, especially since the WHO released its second version in 2025, which added three key factors in older people’s health: urinary incontinence, carer support, social care and support domains [16–18].

Aligned with this approach, the WHO also launched the Decade of Healthy Ageing (2021–2030) with the goal of reducing health inequalities and improving the lives of older people, their families, and communities [12]. Several authors have hypothesized that intrinsic capacity assessed by these tools, may help identify individuals at higher risk of developing frailty, functional decline, and adverse health outcomes. If confirmed, these tools could be integrated into clinical practice for community-dwelling older adults, encouraging greater involvement in the management of their own health and improving self-care [19, 20].

In the context of an ageing population and the increasing burden of frailty, dependency, and disability, ensuring the training of future health professionals becomes more critical than ever. Preparing students in health-related fields to understand the complexities of ageing, promote healthy lifestyles, provide person-centred and interdisciplinary care is essential to meet the growing needs of older adults. Equipping future professionals with knowledge, empathy, and practical skills ensures their capability of addressing both the medical and social aspects of ageing. Knowledge about aging and older adults among undergraduate health students remains insufficient, as various studies indicate that they often hold negative perceptions about this population [21, 22]. In addition, to conduct a proper assessment, the attitudes of healthcare professionals and future professionals must be considered. Age is a determinant of health and is connected to ageism. Although it is present systemically in healthcare, characterizing and understanding the complex factors influencing ageism can positively impact the lives and health outcomes of older adults [23]. Increasing this knowledge has been identified as an effective strategy to reduce these negative attitudes [24]. Moreover, early exposure to real-world settings and vulnerable populations can foster a stronger commitment to geriatric care, ultimately contributing to more resilient and responsive health and social systems. It has been observed that the earlier young students have contact with older adults, the better their attitude towards this group [25].

The Service-Learning methodology is used as a framework, as it is an innovative teaching strategy through which students learn by participating in an intervention aimed at addressing a real community need. Through Service-Learning, students offer meaningful contributions to the community by engaging in direct, hands-on interaction. Several studies in the literature describe Service-Learning experiences involving students and older adults, such as telecollaborative projects during the COVID-19 pandemic, intergenerational programs focused on dementia, fall prevention initiatives, and interprofessional interventions like music and memory. These experiences consistently highlight the benefits of intergenerational learning, professional development, and improved attitudes toward aging [26–34]. However, none of the reviewed studies explicitly incorporated a CGA as part of their Service-Learning activities. This project therefore represents a novel intersection between two well-established but rarely integrated frameworks: CGA as a clinical tool, and Service-Learning as a pedagogical methodology. Highlighting this unique contribution more explicitly could help clarify the originality and added value of the study. One study evaluating updates to CGA emphasizes that integrating CGA into the care of older adults is crucial. The study highlights the relevance and necessity of including specific and thorough training on how to perform a CGA in undergraduate medical and nursing education [35].

In the current context of population aging, the prevention and promotion of health among older adults are crucial for achieving autonomy and improving quality of life. Although ICOPE has already been tested and explored in other European countries, such as France and Andorra [19, 36, 37], only one study has been identified in Catalonia, Spain where such testing has been conducted [38]. Furthermore, the training of future professionals to care for older adults is essential in the actual context. Service-Learning represents an innovative educational approach that strengthens students’ academic, professional, and civic competencies while promoting social responsibility and community engagement. In this regard, universities can play a key role as community health assets, acting as facilitators of intergenerational learning and health promotion. By linking education with real community needs, this type of initiative contributes to addressing the challenges of population ageing and to fostering healthier, more inclusive societies.

## Research aims

The general objective of this study is to evaluate the impact of a Service-Learning program that brings undergraduate health students into contact with community-dwelling older adults, assessing its benefits for students’ professional development and for older adults’ knowledge regarding their health.

Based on this general objective, the study addresses several specific goals for both students and older adults that operationalize the evaluation of the programme’s impact.

### Students

- Analyse undergraduate health students’ perceptions of their professional training through the Service-Learning methodology, focusing on three interrelated dimensions: the formative development as an educational goal, the learning process as a means, and the service component as a commitment to the community.
- Identify distinct student profiles based on their responses to the Service-Learning questionnaire through a cluster analysis, and to examine how these profiles differ according to sociodemographic variables: age, sex, and university degree.
- Describe the impact of the Service-Learning intervention on soft skills: communication, interdisciplinary teamwork, and critical thinking.
- Describe the impact of the Service-Learning intervention on specific competencies related to Comprehensive Geriatric Assessment and Intrinsic Capacity.
- Compare students’ satisfaction levels, competences, and soft skills across different sessions.

### Users (≥ 60 years old)

- Describe the characteristics of older adults including the prevalence of frailty and losses of intrinsic capacity.
- Identify the possible association between the ICOPE framework and VIG-Frail in individuals aged 60 and older.
- Investigate the possible association between the ICOPE and selected Nursing Outcomes Classification (NOC) indicators in individuals aged 60 and older.
- Analyse the impact of the program on their knowledge of their health status, lifestyle, risk factors of chronic conditions, community-based health assets and satisfaction.

## Methods/design

### Aim, design and setting of the study

The study was registered in *Clinical Trials* with the registration number NCT06890325 on March 24, 2025 (https://clinicaltrials.gov/study/NCT06890325). It is a one-arm intervention study that employs a single-centre mixed-methods approach, following the experimental mixed-methods framework outlined by Creswell and Creswell (2018) [39]. The study aims to analyse undergraduate health students’ perceptions of their professional training through the Service-Learning methodology, focusing on three dimensions: formative, learning, and service. This community-based educational approach also contributes to enhancing health knowledge among participants aged 60 years and older.

The study will be carried out in the Osona region (Barcelona, Spain), at the University of Vic-Central University of Catalonia (UVic-UCC), in collaboration with various community centres including primary care centres, neighbourhood associations and social organisations within the local community, such as Equip d’Assitència Primària Vic - CAP El Remei Vic sud, ASHES – Associació per a la Sostenibilitat Humana, Ecològica i Social, the Associació de Veïns Horta Vermella, and the Germanetes dels Pobres de Vic.

### Characteristics of participants or description of materials

The study comprises two population groups: university students and older adults. Students will be recruited from five undergraduate programs at UVic-UCC (Nursing, Physiotherapy, Human Nutrition and Dietetics, Occupational Therapy, and Psychology), specifically those enrolled in courses focused on ageing-related topics.

Participants aged 60 and above who can travel independently or with caregiver support to the UVic-UCC facilities will be eligible to participate. Individuals presenting cognitive impairment or significant communication difficulties will be excluded to ensure adequate understanding and participation in the intervention. Throughout the study, participants will remain under their usual care, since the intervention is designed to be compatible with and not a substitute for standard treatment. Recruitment will be through key associations and institutions in the Osona region. Snowball sampling and social media will also be used for participant enrolment.

The sampling performed is non-probabilistic and either consecutive or convenience-based, according to the criteria described above. The project will be presented at various community associations. Regarding the students, they will be recruited through the participating courses of the previously mentioned university degree programs.

### Description of all processes, interventions, and comparisons

#### Structure of the intervention

**Fig. 1:**
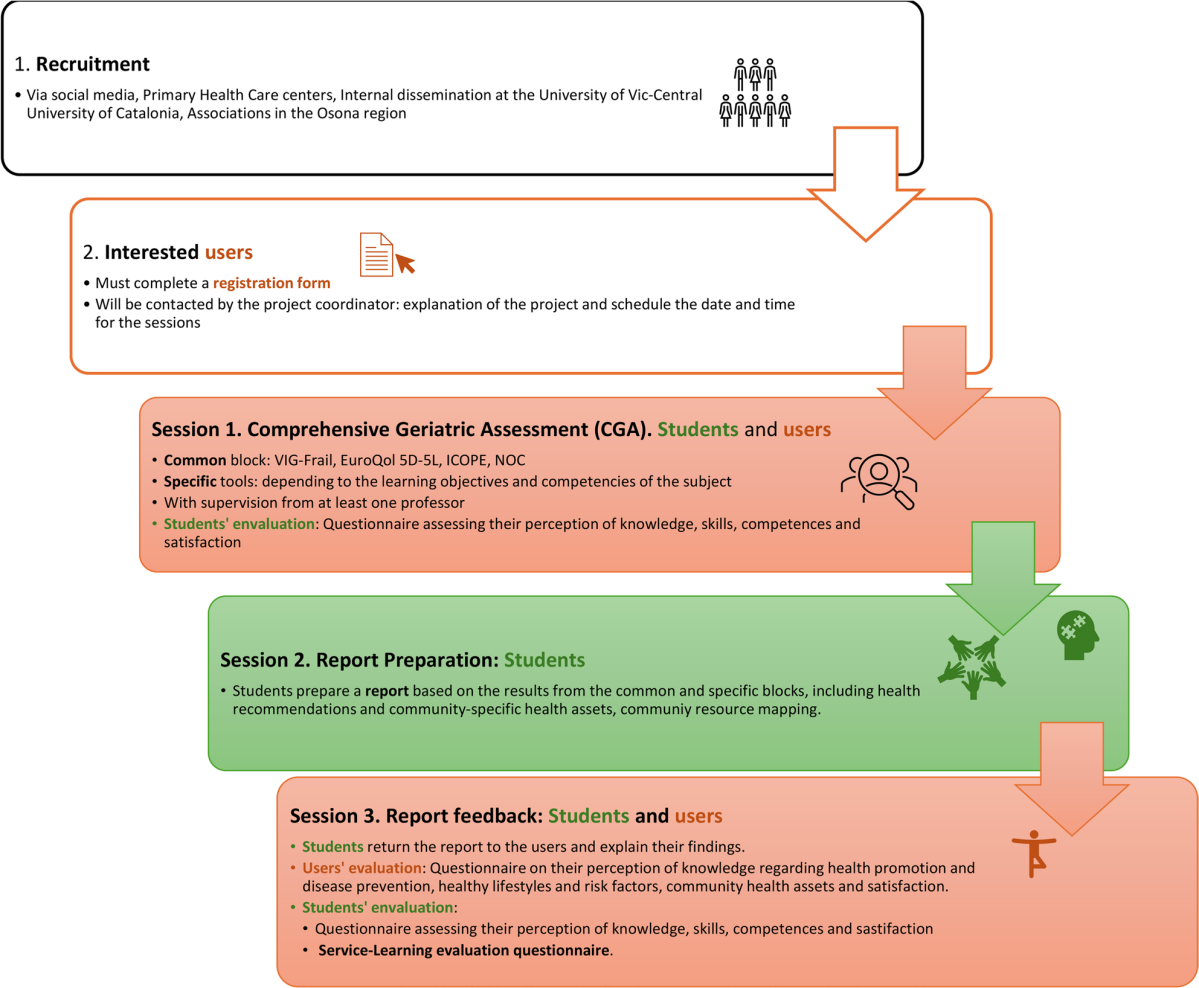
Description of the intervention process

The intervention will consist of three sessions, during which older adult participants will come to the university (Fig. 1). During the first visit, all older adults will meet at a designated meeting point with the professor, also the purpose and procedures of the intervention will be reviewed, and participants will be invited to participate in the study and asked to sign the informed consent form. Approximately 12–20 students will be present in the classroom, divided into groups of up to 5 students per each older adult as stated in Order SSI/81/2017 published in the Spanish Official State Gazette (BOE). A maximum ratio of five students per user is established in order to prevent an excessive number of students per participant, thereby ensuring the quality of learning and, most importantly, safeguarding the patient’s right to privacy and comfort [40].

The welcome session will last approximately 5 to 10 min, after which each group will be guided to their assigned classroom. The first session will include a common assessment block based on CGA lasting 20 to 30 min. This block includes the collection of sociodemographic data (age, gender, education level, neighbourhood/area of residence, whether the participant requires a caregiver or is a caregiver, last occupation, and current living situation). Participants will also be asked about their preferences, values, priorities, and social context, as well as their expectations for the intervention.

Within this common block, various tests will be administered: ICOPE, VIG-Frail, EuroQol-5D-5L and indicators of Nursing Outcomes Classification (NOC). Following this, a specific assessment block will last approximately 5 to 10 min. This specific block will include a questionnaire or test aligned with the learning objectives and competencies of each subject, which may vary depending on the subject participating in the project. The data collected in this subject-specific block will not be used for research purposes; it will be used exclusively by the students to complete their reports. After each session, participants will be dismissed, and a debriefing session will be held with the students.

The next session will consist of a classroom-based student activity (without the older adults). During this session, students will prepare a report based on the CGA and will develop personalized health recommendations for the older adult, including health education strategies and suggestions for referral to appropriate community health assets and programs. The report will include: (1) a summary table of the common block results and key domains: frailty (VIG-Frail score), quality of life (EUROQOL-5D-5L), cognition, mobility, nutrition, vision, hearing, psychological status, social support, caregiver status, and urinary incontinence-interpreted based on the ICOPE and NOC responses; (2) a section on the (subject-depending) specific block, detailing the test used, its purpose, and results; and (3) a section with personalized health promotion and prevention recommendations, including community-based health resources aligned with the participant’s preferences and place of residence. All reports will be reviewed by the course professor and the project coordinator. This dual review process helps ensure the quality and consistency of the content before it is shared with the users.

The final session will include the visit from older adults. To improve adherence to the intervention plan, reminder phone calls will be made to participants before this session. During this visit, students will meet again at the university with the same older adults they assessed during the first visit and present the personalized health report prepared in class. The main objective of this session will be for students to explain their findings and recommendations, applying personalized recommendations to the specific case of the older adult. In cases where the participant is unable to attend the session in person, data collection will be conducted via telephone.

At the end of the intervention of each semester, focus groups with students, professors, and older adults will be conducted to explore their experiences and perceptions of the programme. Further details on the procedures and data collection methods for these focus groups are provided in the Data Collection Methods section.

#### Data collection methods

The instruments included in the study are presented in two distinct blocks: one referring to the tools administered to students and another to those applied to the older adult participants.

##### Students

A validated questionnaire developed by León-Carrascosa et al. (2020) is used to evaluate the students’ perceptions of the Service‑Learning methodology in higher education. The questionnaire consists of 29 items distributed across three dimensions that reflect the core components of Service-Learning: the formative aspect as an educational goal, the learning process as a means, and the service component as a commitment to the community. Each item is rated on a Likert-type scale ranging from 1 (strongly disagree) to 5 (strongly agree). The instrument has demonstrated adequate psychometric properties in terms of validity and reliability and has been previously applied in university contexts related to health and social education. The questionnaire will be self-administered by the students at the end of the third session.

An additional questionnaire was specifically developed for this research to assess students’ perceived acquisition of knowledge and competencies related to health promotion, disease prevention, CGA, and Intrinsic Capacity. It also includes items addressing transversal skills such as critical thinking, and problem solving, as well as perceived integration of knowledge and overall satisfaction with the project. Items were formulated in neutral language to minimise response bias related to positively or negatively loaded wording. The questionnaire will be self-administered at the end of the first and last sessions. As each session focuses on a different learning domain, the two assessments correspond to distinct sets of competencies rather than repeated measures of the same construct. Detailed items can be found in the supplementary material.

##### Older adults

A specific questionnaire was also developed for community dwelling older adults to evaluate their perceived knowledge of their own health status, their awareness of lifestyle habits and modifiable risk factors, their understanding of community health resources and assets, and their overall satisfaction with the experience. This questionnaire will be completed at the end of the final session. Detailed questions are available in the supplementary file.

All items for both target populations will be rated on a 5-point Likert scale (1 = “not at all” to 5 = “very much”). All questionnaires will be created and administered through the REDCap platform. Research team members have undergone prior training to ensure their standardized administration and the accuracy of data collection.

In addition to the self-reported questionnaire, several validated instruments will be used to assess the health status of the older adults. The instruments include ICOPE tool to assess intrinsic capacity [16], the validated VIG-Frail scale to evaluate frailty status [41], the EuroQol-5D-5L to measure quality of life and eighteen indicators of NOC. The EuroQol-5D-5L is a widely used instrument validated for measuring self-perceived health and quality of life and has normative reference values for the Spanish population [42, 43].

The NOC was developed in 1991 at the University of Iowa following research involving professionals from various specialties, as a standardized grouping of outcomes obtained in nursing clinical practice. It enables the identification, naming, classification, and measurement of outcomes and indicators achievable through Nursing Interventions Classification (NIC), based on the profession’s own common Language [44]. The NOC is useful across all areas of nursing practice but can also be used by other healthcare professionals, as it describes the condition of patients, caregivers, families, or communities at a conceptual level and provides a classification of user outcomes that may be influenced by all healthcare disciplines [45]. Each NOC includes an identification number, a definition, and a set of indicators that describe specific states, perceptions, or behaviours related to the outcome, measured using a 5-point Likert scale (1 indicating severe and 5 indicating absence). The outcome helps healthcare professionals assess and quantify the condition of the patient, caregiver, family, or community [46]. The aim is to relate the nine priority conditions associated with the decline of intrinsic capacity according to ICOPE to nine NOCs, selecting two indicators for each NOC. In doing so, an added value will be provided to the ICOPE instrument, exploring the consistency and potential complementarity of both assessment approaches, as no study has yet been found that establishes this relationship. This innovative association between ICOPE and NOC aims to open new avenues for integrated assessment models in geriatric care, potentially improving the interdisciplinary understanding of functional decline. Exploring such correlations may also inform the development of shared care plans across professional domains.

As part of the qualitative data collection, focus groups will be conducted with all potential participants (students, professors, and older adults), who will be informed and invited to participate voluntarily at the end of each semester. Separate focus groups will be organised for students and professors, ensuring representation from the different undergraduate health degrees involved in the programme. These sessions will explore how they experienced their involvement in the Service‑Learning programme, whether it influenced their knowledge and competencies, and their perceptions of the programme’s value and areas for improvement. In parallel, focus groups will also be conducted with older adult participants to explore how the programme has affected their knowledge about their own health status, whether they have learned strategies to improve their health, and whether their awareness of community health resources has increased. All focus groups will be conducted online via Microsoft Teams, and sessions will be recorded to support accurate transcription. Details on the coding and thematic procedures for qualitative analysis are provided in the Statistical Analysis section.

### Statistical analysis

Descriptive statistics will be calculated for all study variables. Continuous variables will be summarised as mean and standard deviation for normally distributed data, or median and interquartile range for non-normally distributed data. Categorical variables will be expressed as absolute and relative frequencies (n, %). Normality will be assessed using the Kolmogorov–Smirnov test and visual inspection of histograms and Q–Q plots.

Mean scores for each dimension will be computed, and potential differences between degree programmes will be analysed using one-way ANOVA or Kruskal–Wallis tests, depending on data distribution.

A cluster analysis will be conducted to identify distinct student profiles based on their responses to the 29-item Service-Learning questionnaire covering the three dimensions of training, learning, and service. The K-means method will be applied, as it allows grouping students according to the similarity of their response patterns. Different values of *k* will initially be explored to determine the most meaningful and interpretable number of clusters. The final solution will be selected based on the internal coherence of the clusters and the clarity of their conceptual interpretation.

Once the final clusters are obtained, each group will be characterised by examining the mean scores of the questionnaire items or dimensions. This will allow the identification of distinctive patterns of perception and applicability of the Service-Learning methodology within each profile.

To examine how these profiles differ according to sociodemographic variables, subsequent analyses will be performed. Differences in age across clusters will be evaluated using ANOVA or the Kruskal–Wallis test when assumptions of normality and homogeneity of variances are not met. Associations between clusters and categorical variables such as sex and university degree will be assessed using chi-square tests.

A significance level of *p* < 0.05 will be applied to all statistical tests.

At baseline, univariate associations between ICOPE, VIG-Frail, and the components of the NOC outcomes will be explored. To examine linear relationships between continuous variables, Pearson’s correlation coefficients will be calculated when normality assumptions are met; otherwise, Spearman’s rank correlation coefficients will be used. Statistical significance will be set at *p* < 0.05. When multiple comparisons are performed, Bonferroni correction will be applied to adjust the significance threshold accordingly. No imputation methods will be applied for missing data, only complete-case analysis will be performed. The quantitative data will be analysed using SPSS software (version 29.0).

Qualitative data from focus groups will be conducted online via Teams. Participants will sign an authorization for recording and image use. The analysis process will be conducted by using a modified version of Colaizzi’s 7-stage method, which draws upon Husserl’s descriptive phenomenological approach that aims to describe the essence of lived experiences from the first-person perspective. In-depth thematic analysis will be finalized [47]. Adherence to the Standards for Reporting Qualitative Research will be ensured throughout the study [48].

A Data Monitoring Committee (DMC) has been established, consisting of the Principal Investigator, the study coordinator, one expert in data analysis, and one expert in the data collection platform (REDCap). The DMC will conduct periodic reviews of the intervention to ensure its adherence and data quality and analysis. Also, will monitor study progress and would be responsible for any decision regarding early termination if unforeseen circumstances arise.

### Power calculation

An initial sample size will be considered, and iterative calculations will be performed until adequate statistical power is achieved. As a reference point, we will start from the figure of 304 participants reported in the literature for studies in the field of social sciences [49]. However, since our research area differs substantially and is focused on health sciences, this initial value will be adjusted accordingly based on the specific characteristics and requirements of the study.

## Discussion

This project is expected to generate impact at several levels: (1) undergraduate health students; (2) older adults; (3) teaching and research staff; and (4) a broader meso and macro level, including health and community institutions or services, as well as territorial or scientific dimensions.

At the student level, the intervention is expected to enhance knowledge in CGA (thorough assessment instruments), health promotion, and disease prevention, and apply theoretical knowledge learned in class to real-life situations. The administration of the Service-Learning evaluation questionnaire provides a comprehensive view of how students perceive their development across the formative, learning, and service dimensions, offering valuable insight into the added value of Service-Learning beyond traditional academic instruction.

Through the Service-Learning approach, students not only strengthen their technical competencies but also engage in meaningful interactions with older adults, fostering reflective practice, social awareness, and a sense of civic responsibility. This experiential component is a defining feature of Service-Learning and represents a crucial educational opportunity to integrate knowledge, skills, and attitudes in a real community context.

According to Dahlke et al. (2020) [50], meaningful learning experiences with older adults in various contexts are necessary to carefully plan how to disrupt negative perceptions that may arise during undergraduate training. Experiential education opportunities that allow university students to interact positively with older adults can help replace negative age-related beliefs [21]. Furthermore, improvements are expected in interpersonal and intrapersonal communication skills, critical thinking, problem-solving abilities, and interdisciplinary teamwork. The aim is to prepare the next generation of leaders in health-related fields, professionals capable of responding to the rapid and complex evolution of social and healthcare systems, in alignment with the integrated care model promoted by the Departments of Social Rights and Health of the Government of Catalonia.

For the older adult participants, the intervention will serve as a valuable source of feedback on their current health status. They will have the opportunity to learn healthy habits and lifestyles that support an improvement in their quality of life, contributing to active and healthy ageing. They also benefit from interaction with students, which stimulates their mental and emotional activity and may help reduce feelings of social isolation. Engaging in dialogue and sharing personal experiences can further strengthen their self-esteem and sense of belonging [51, 52].

Connecting undergraduate health students with older adults creates a highly positive impact on the community, as it promotes the formation of meaningful bonds that support mutual learning and intergenerational understanding. This approach clearly aligns with several Sustainable Development Goals (SDGs) of the 2030 Agenda, particularly SDG 3 (Good Health and Well-being), SDG 4 (Quality Education), and SDG 10 (Reduced Inequalities), promoting a more just, inclusive, and healthy society through citizen empowerment and the social responsibility of future healthcare professionals. Several studies agree that it is helpful both for the students who take part and for the people or communities who benefit from these projects [53]. Students often feel more confident and capable, and the chance to reflect on their experience helps them understand their own strengths and areas for improvement. It also allows them to connect what they learn in class with real-life situations [54]. In the United States, Service-Learning has also shown positive results in increasing students’ awareness of social issues and improving their attitudes towards older adults [55, 56] while simultaneously integrating course content with real-world experience [57]. In Europe, other studies have shown similar positive results in Service-Learning (SL) projects, where community engagement not only enhances academic outcomes and improves competences, such as “developing as health professionals”, but also fosters a sense of belonging and social responsibility contributing to students’ professional growth [58–62]. This project has the potential to facilitate early detection of frailty, promote healthy ageing, and strengthen connections between universities, primary care, and local resources.

From the perspective of the teaching staff involved, the project will foster the acquisition of new knowledge and the development of competencies such as interdisciplinary collaboration, specific skills in CGA, and the promotion of health and disease prevention. It will also support the development of effective teamwork and help define best practices in conducting comprehensive geriatric assessments.

At the meso and macro level university-wide, territorial, and scientific, the project will raise the visibility of the university facilities contributing to the university’s integration into the local territory. Partnerships with key health and social care institutions, will be strengthened. Results of the study will be disseminated through scientific publications, presentations at conferences on health and education, and communication via institutional and social media channels to reach both academic and community audiences. National and international dissemination activities will position the CGA workshop as a model that could be replicated by other institutions across Catalonia and beyond. This model could be adapted and transferred to other contexts with fewer resources or different demographic characteristics, such as rural areas or regions with limited access to geriatric care. Evaluating the model’s effectiveness in diverse settings would also be essential to guide adaptation. In addition, one of the potential benefits of this type of initiative is the reduction of healthcare system costs. By improving the training of future professionals and empowering older adults through more integrated and person-centered care, early detection and prevention strategies are reinforced, potentially decreasing the need for more complex or long-term interventions. This economic dimension supports the relevance and scalability of the model in both well-resourced and constrained settings. Overall, the project may contribute to preparing future professionals for the challenges of population ageing while positioning the university as an active agent in community well-being.

One of the strengths of this project is its innovative application of Service-Learning methodology to CGA and the measurement of intrinsic capacity involving undergraduate students interdisciplinary across different health-care fields. Moreover, it represents one of the few studies using the ICOPE tool worldwide. Notably, no previous initiatives using ICOPE have been reported in the Central Catalonia region. VIG-Frail has demonstrated both convergent and discriminant validity when compared with other instruments, such as the EuroQol-5D-5 L for quality of life and the Braden Scale for pressure injury risk. However, to date, no study has yet examined its correlation with ICOPE [63, 64]. A major strength of this study is the use of the validated questionnaire developed by León-Carrascosa et al. [65] which has not previously been applied to such a large and diverse sample of undergraduate health students. This will provide valuable evidence on the performance and generalisability of the instrument in the context of health-related education. Furthermore, no previous Service-Learning initiatives incorporating a CGA as a core component have been identified in the published literature, underscoring the innovative contribution of this project.

Compared to similar innovations, this project stands out for its strong integration between the community and the university environment. Previous studies have shown positive outcomes in Service-Learning projects, where community engagement not only enhances academic performance but also fosters a sense of belonging and social responsibility [58]. Also, this innovative association between ICOPE and NOC aims to open new avenues for integrated assessment models in geriatric care, potentially improving the interdisciplinary understanding of functional decline.

For the limitations, a risk management plan has been developed to address them. One anticipated challenge is participant recruitment, which may be mitigated through collaboration with key community organizations or by offering visits at user locations. Another potential limitation is the non-attendance of older adults on session days; this may be addressed by converting the session into a role-play, practical workshop, clinical case discussion, or report preparation. The absence of a control group may limit the evaluation of intervention effectiveness. On the other hand, the specific objectives of the assessment of students’ soft skills and older adults’ knowledge is based on self-perceived evaluations, which may limit the validity of the results. Additionally, no pre-post test will be administered, which could reduce the reliability of the findings. No imputation methods will be applied for missing data, only complete-case analysis will be performed, acknowledging that this approach may reduce the statistical power of the results. Lastly, logistical challenges may arise, such as managing space, scheduling, and ensuring multidisciplinary interaction among student groups.

## Supplementary Information

Supplementary Material 1.

Supplementary Material 2.

## Data Availability

The data and materials generated and analysed during the current study are available from the corresponding author upon explicit request, to promote transparency and collaborative research.

## Abbreviations

CGA: Comprehensive Geriatric Assessment
ICOPE: Integrated Care for Older People
NOC: Nursing Outcomes Classification
UVic-UCC: University of Vic – Central University of Catalonia
WHO: World Health Organization

## References

[1] World Health Organization. Ageing. Available from: https://www.who.int/news-room/facts-inpictures/detail/ageing. [publisehd 2018; cited 2025 Apr 24].

[2] Institut d’Estadística de Catalunya. (Idescat). Indicadors demogràfics i de territori. Estructura per edats, envelliment i dependència. Catalunya. Available from: https://www.idescat.cat/pub/?id=inddt&n=915&geo=cat. [updated 2025; cited 2024 July 25].

[3] Institut d’Estadística de Catalunya. Idescat. Novetats. Taula de vida 2018–2022. Available from: https://www.idescat.cat/novetats/?id=5131. [published 2025; cited 2025 Feb 24].

[4] Instituto Nacional de Estadística (INE). INEbase. Demografía y población. Cifras de población y Censos demográficos. Proyecciones de población. Últimos datos. Available from: https://www.ine.es/dyngs/INEbase/es/operacion.htm?c=Estadistica_C&cid=1254736176953&menu=ultiDatos&idp=1254735572981. [published 2024; cited 2024 Jul 25].

[5] Institut d’Estadística de Catalunya (Idescat). Novetats. Estimacions de població. S2/2023-S1/2024. Dades definitives. Available from: https://www.idescat.cat/novetats/?id=5124. [published 2025; cited 2025 Feb 24].

[6] World Health Organization (WHO). Se calcula que el número de personas mayores de 60 años se duplicará de aquí a 2050. Available from: https://www.who.int/es/news/item/30-09-2015-who-number-of-people-over-60-years-set-to-double-by-2050-major-societal-changes-required. [published 2015; cited 2024 Aug 1].

[7] Observatori Socioeconòmic d’Osona. L’atur registrat a Osona augmenta al març per segon mes consecutiu. Available from: https://www.observatorisocioeconomicosona.cat/index.php?seccio=destacats&id=254. [published 2025; cited 2025 Apr 24].

[8] Diputació de Barcelona. Informe econòmic local de la província de Barcelona. Osona. Available from: https://www.diba.cat/ca/web/informe-economic-local-de-la-provincia-de-barcelona/2024/osona. [cited 2025 Apr 24].

[9] Feng Z, Lugtenberg M, Franse C, Fang X, Hu S, Jin C et al. Risk factors and protective factors associated with incident or increase of frailty among community-dwelling older adults: A systematic review of longitudinal studies. PLoS ONE. 2017;12(6):e0178383. Available from: https://journals.plos.org/plosone/article?id=10.1371/journal.pone.0178383. [cited 2025 Apr 24]. 10.1371/journal.pone.0178383 PMC5472269 28617837

[10] O’Caoimh R, Galluzzo L, Rodríguez-Laso Á, Van der Heyden J, Ranhoff AH, Lamprini-Koula M, et al. Prevalence of frailty at population level in European ADVANTAGE Joint Action Member States: a systematic review and meta-analysis. Ann Ist Super Sanita. 2018;54(3):226–38. 10.4415/ann_18_03_10. 30284550 10.4415/ANN_18_03_10

[11] Associació d’Infermeria Familiar i Comunitària de Catalunya (AIFiCC). Guia pràctica d’atenció a la fragilitat des de l’atenció primària i comunitària [Internet]. Barcelona: AIFiCC. 2022. Available from: https://www.aificc.cat/wp-content/uploads/2022/07/Fragilitat_AIFiCC_2022.pdf. [cited 2024 Aug 1].

[12] World Health Organization (WHO). Década de envejecimiento saludable 2020–2030. Available from: https://www.who.int/es/publications/m/item/decade-of-healthy-ageing-plan-of-action. [published 2020 Des 14; cited 2024 Aug 13].

[13] Lee H, Lee E, Jang IY. Frailty and Comprehensive Geriatric Assessment. J Korean Med Sci. 2019;35(3). 10.3346/jkms.2020.35.e16. 10.3346/jkms.2020.35.e16 PMC6970074 31950775

[14] Stuck AE, Aronow HU, Steiner A, Alessi CA, Büla CJ, Gold MN, et al. A Trial of Annual in-Home Comprehensive Geriatric Assessments for Elderly People Living in the Community. N Engl J Med. 1995;333(18):1184–9. 10.1056/NEJM199511023331805. 7565974 10.1056/NEJM199511023331805

[15] Cesari M, Araujo de Carvalho I, Amuthavalli Thiyagarajan J, Cooper C, Martin FC, Reginster JY, et al. Evidence for the Domains Supporting the Construct of Intrinsic Capacity. J Gerontol Ser A. 2018;73(12):1653–60. 10.1093/gerona/gly011 29408961

[16] World Health Organization. Integrated care for older people (ICOPE): guidance for person-centred assessment and pathways in primary care. Second edition. Geneva: World Health Organization. 2025. Available from: https://iris.who.int/handle/10665/250239. [cited 25 febrero 2025].

[17] World Health Organization. Integrated care for older people (ICOPE): guidance for person-centred assessment and pathways in primary care. 2019. Available from: https://iris.who.int/handle/10665/326843. [cited 2024 Aug 13].

[18] World Health Organization. Atención integrada para las personas mayores (ICOPE): Guía sobre la evaluación y los esquemas de atención centrados en la persona en la atención primaria de salud. Manual - OPS/OMS | Organización Panamericana de la Salud. 2020. Available from: https://www.paho.org/es/documentos/atencion-integrada-para-personas-mayores-icope-guia-sobre-evaluacion-esquemas-atencion. [cited 2024 Aug 13].

[19] Sanchez-Rodriguez D, Piccard S, Dardenne N, Giet D, Annweiler C, Gillain S. Implementation of the Integrated Care of Older People (ICOPE) App and ICOPE Monitor in Primary Care: A study protocol. J Frailty Aging. 2021;10(3):290–6. 10.14283/jfa.2021.22. 34105715 10.14283/jfa.2021.22

[20] Abades Porcel M, Rayón Valpuesta E. El envejecimiento en España: ¿un reto o problema social? Gerokomos. 2012;23(4):151–5. 10.4321/S1134-928X2012000400002.

[21] Newsham TMK, Schuster AM, Guest MA, Nikzad-Terhune K, Rowles GD. College students’ perceptions of old people compared to grandparents. Educ Gerontol. 2021;47(2):63–71. 10.1080/03601277.2020.1856918.

[22] Ekwonye AU, Malek A, Farah I, Nguyen S, Chonyi T, Ponce-Diaz V, et al. Aging is beautiful and graceful: Exploring college students’ perceptions of aging, older adults, and future older selves. Educ Gerontol. 2023;49(9):803–16. 10.1080/03601277.2022.2164642. 37942281 10.1080/03601277.2022.2164642 PMC10629836

[23] Palsgaard P, Maino Vieytes CA, Peterson N, Francis SL, Monroe-Lord L, Sahyoun NR, et al. Healthcare Professionals’ Views and Perspectives towards Aging. Int J Environ Res Public Health. 2022;19(23):15870. 10.3390/ijerph192315870. 36497945 10.3390/ijerph192315870 PMC9739620

[24] Allan LJ, Johnson JA. Undergraduate Attitudes Toward the Elderly: The Role of Knowledge, Contact and Aging Anxiety. Educ Gerontol. 2008;35(1):1–14. 10.1080/03601270802299780.

[25] Martina M, Gutiérrez C, Mejia M, Terukina R. Percepción del estudiante de medicina de una universidad pública acerca del docente adulto mayor y del adulto mayor en general. Fac Med. 2014 July;75(3):237–44. 10.15381/anales.v75i3.9777.

[26] Meuser T, Cohen Konrad S, Robnett R, Brooks F. Telecollaboration in gerontology service learning: Addressing isolation & loneliness in a pandemic. Gerontol Geriatr Educ. 2022;43(1):18–33. 10.1080/02701960.2021.1956489. 34348587 10.1080/02701960.2021.1956489

[27] Yamashita T, Kinney JM, Lokon EJ. The impact of a gerontology course and a service-learning program on college students’ attitudes toward people with dementia. J Appl Gerontol Off J South Gerontol Soc. 2013;32(2):139–63. 10.1080/02701960.2021.1956489. 10.1177/0733464811405198 25474214

[28] Horowitz BP, Wong SD, Dechello K. Intergenerational service learning: to promote active aging, and occupational therapy gerontology practice. Gerontol Geriatr Educ. 2010;31(1):75–91. 10.1080/02701960903578345. 20390628 10.1080/02701960903578345

[29] Kimbler KJ, Ehman AC. Gerontology and youth-focused service learning: the relation between service recipient age and student responses. Gerontol Geriatr Educ. 2015;36(4):384–95. 10.1080/02701960.2014.925890. 24884577 10.1080/02701960.2014.925890

[30] Long EM, Gummelt G. Experiential Service Learning: Building skills and sensitivity with Kolb’s learning theory. Gerontol Geriatr Educ. 2020;41(2):219–32. 10.1080/02701960.2019.1673386. 31564226 10.1080/02701960.2019.1673386

[31] Roodin P, Brown LH, Shedlock D. Intergenerational service-learning: a review of recent literature and directions for the future. Gerontol Geriatr Educ. 2013;34(1):3–25. 10.1080/02701960.2012.755624. 23362852 10.1080/02701960.2012.755624

[32] Sum G, Lau LK, Jabbar KA, Lun P, George PP, Munro YL, et al. The World Health Organization (WHO) Integrated Care for Older People (ICOPE) Framework: A Narrative Review on Its Adoption Worldwide and Lessons Learnt. Int J Environ Res Public Health. 2022;20(1):154. 10.3390/ijerph20010154. 36612480 10.3390/ijerph20010154 PMC9819593

[33] Cannon ML, Perkinson MA, DeLaTorre AK, Martinez IL, Ozer E, Sweatman WM et al. Service-Learning through conference-based, interdisciplinary workshops on Age-Friendly design. Gerontol Geriatr Educ. 2020;41(2):200–5. 10.1080/02701960.2019.1643337. 10.1080/02701960.2019.1643337 31311490

[34] Laks J, Wilson LA, Khandelwal C, Footman E, Jamison M, Roberts E. Service-Learning in Communities of Elders (SLICE): Development and Evaluation of an Introductory Geriatrics Course for Medical Students. Teach Learn Med. 2016;28(2):210–8. 10.1080/10401334.2016.1146602. 27064723 10.1080/10401334.2016.1146602

[35] Hospital Universitario Ramón y Cajal (IRYCIS), Madrid, Sánchez-García E, Montero-Errasquin B, Hospital Universitario Ramón y Cajal (IRYCIS)., Madrid, Cruz-Jentoft A. Hospital Universitario Ramón y Cajal (IRYCIS). Madrid. Comprehensive geriatric assessment: an update. An RANM. 2020;137(01):77–82. 10.32440/ar.2020.137.01.doc01.

[36] Tavassoli N, Piau A, Berbon C, De Kerimel J, Lafont C, De Souto Barreto P, et al. Framework Implementation of the INSPIRE ICOPE-CARE Program in Collaboration with the World Health Organization (WHO) in the Occitania Region. J Frailty Aging. 2021;10(2):103–9. 10.14283/jfa.2020.26. 33575698 10.14283/jfa.2020.26

[37] WHO clinical consortium on healthy ageing 2022. report of consortium meeting, 5–6 December 2022. Available from: https://www.who.int/publications/i/item/9789240076822. [cited 2025 Nov 15].

[38] Rojano I, Luque X, Blancafort-Alias S, Prat Casanovas S, Forné S, Martín Vergara N, Fabregat Povill P, et al. Identification of decreased intrinsic capacity: Performance of diagnostic measures of the ICOPE Screening tool in community dwelling older people in the VIMCI study. BMC Geriatr. 2023;23(1):106. 10.1186/s12877-023-03799-0. 36809987 10.1186/s12877-023-03799-0 PMC9945724

[39] Creswell JW, Creswell JD. Research design: qualitative, quantitative, and mixed methods approaches. Fifth edition. Los Angeles; London: SAGE; 2018. p.275.

[40] de Sanidad M, Servicios Sociales e Igualdad. Orden SSI/81/2017, de 19 de enero, por la que se publica el Acuerdo de la Comisión de Recursos Humanos del Sistema Nacional de Salud, por el que se aprueba el protocolo mediante el que se determinan pautas básicas destinadas a asegurar y proteger el derecho a la intimidad del paciente por los alumnos y residentes en Ciencias de la Salud. Section 3, Orden SSI/81/2017. 2017 p.8277–89. Available from: https://www.boe.es/eli/es/o/2017/01/19/ssi81.

[41] Torné A, Puigoriol E, Zabaleta-del-Olmo E, Zamora-Sánchez JJ, Santaeugènia S, Amblàs-Novellas J, Reliability. Validity, and Feasibility of the Frail-VIG Index. Int J Environ Res Public Health. 2021;18(10):5187. 10.3390/ijerph18105187. 34068227 10.3390/ijerph18105187 PMC8153117

[42] Hernandez G, Garin O, Pardo Y, Vilagut G, Pont À, Suárez M, et al. Validity of the EQ-5D-5L and reference norms for the Spanish population. Qual Life Res Int J Qual Life Asp Treat Care Rehabil. 2018 Sept;27(9):2337–48. 10.1007/s11136-018-1877-5. 10.1007/s11136-018-1877-5 29767329

[43] Garcia-Gordillo MA, Adsuar JC, Olivares PR. Normative values of EQ-5D-5L: in a Spanish representative population sample from Spanish Health Survey, 2011. Qual Life Res Int J Qual Life Asp Treat Care Rehabil. 2016;25(5):1313–21. 10.1007/s11136-015-1164-7. 10.1007/s11136-015-1164-7 26482825

[44] Correro-Bermejo A, Fernández-Gutiérrez M, Poza-Méndez M, Bas-Sarmiento P. Content and Clinical Validation of the Nursing Outcome Health Literacy Behaviour: A Validation Protocol. Healthcare. 2023;11(4):481. 10.3390/healthcare11040481. 36833015 10.3390/healthcare11040481 PMC9957519

[45] Moorhead S. Clasificación de resultados de enfermería (NOC) : medición de resultados en salud. 7^a^ ed. Clasificación de resultados de enfermería (NOC) : medición de resultados en salud. Barcelona: Elsevier; 2024.

[46] Moorhead Sue. Clasificación de resultados de enfermería (NOC). 4a ed. Clasificación de resultados de enfermería (NOC). Barcelona: Elsevier; 2009.

[47] Wirihana L, Welch A, Williamson M, Christensen M, Bakon S, Craft J. Using Colaizzi’s method of data analysis to explore the experiences of nurse academics teaching on satellite campuses. Nurse Res. 2018;25(4):30–4. 10.7748/nr.2018.e1516. 29546965 10.7748/nr.2018.e1516

[48] Dossett LA, Kaji AH, Cochran A. SRQR and COREQ Reporting Guidelines for Qualitative Studies. JAMA Surg. 2021;156(9):875–6. 10.1001/jamasurg.2021.0525. 10.1001/jamasurg.2021.0525 33825809

[49] Sánchez SS, Belando-Montoro MR, León Carrascosa V. Eficacia percibida y perfiles estudiantiles en el Aprendizaje-Servicio universitario. Rev Educ. 2025;4071–27. 10.4438/1988-592X-RE-2025-407-651.

[50] Dahlke S, Davidson S, Kalogirou MR, Swoboda NL, Hunter KF, Fox MT, et al. Nursing faculty and students’ perspectives of how students learn to work with older people. Nurse Educ Today. 2020;93:104537. 10.1016/j.nedt.2020.104537. 32717698 10.1016/j.nedt.2020.104537

[51] Ruiz-Montero PJ, Chiva-Bartoll O, Salvador-García C, Martín-Moya R. Service-Learning with College Students toward Health-Care of Older Adults: A Systematic Review. Int J Environ Res Public Health. 2019;16(22):4497. 10.3390/ijerph16224497. 31739647 10.3390/ijerph16224497 PMC6888558

[52] Krout JA, Bergman E, Bianconi P, Caldwell K, Dorsey J, Durnford S, et al. Intergenerational service learning with elders: multidisciplinary activities and outcomes. Gerontol Geriatr Educ. 2010;31(1):55–74. 10.1080/02701960903578329. 20390627 10.1080/02701960903578329

[53] Fougère M, Solitander N, Maheshwari S. Achieving Responsible Management Learning Through Enriched Reciprocal Learning: Service-Learning Projects and the Role of Boundary Spanners. J Bus Ethics. 2020;162(4):795–812. 10.1007/s10551-019-04365-8.

[54] Cano EB, García-Martín J. El impacto del aprendizaje-servicio (ApS) en diversas variables psicoeducativas del alumnado universitario: las actitudes cívicas, el pensamiento crítico, las habilidades de trabajo en grupo, la empatía y el autoconcepto. Una revisión sistemática. Rev Complut Educ. 2021;32(4):639–49. 10.5209/rced.70939.

[55] Bowling H, Murray L, Eichler T, Usher B, Fennimore L. Connecting Nursing Students and Older Adults: An Intergenerational Service-Learning Experience. Nurse Educ. 2022;47(1):56. 10.1097/NNE.0000000000001017. 33882532 10.1097/NNE.0000000000001017

[56] Beauvais A, Foito K, Pearlin N, Yost E. Service Learning With a Geriatric Population: Changing Attitudes and Improving Knowledge. Nurse Educ. 2015;40(6):318. 10.1097/NNE.0000000000000181. 25997154 10.1097/NNE.0000000000000181

[57] Hess Brown PAR, Laura. Service-Learning in Gerontology: An Out-of-Classroom Experience. Educ Gerontol. 2001;27(1):89–103. 10.1080/036012701750069067.

[58] Singla FC. L’aprenentatge servei als projectes socioeducatius. Barcelona: Fundació la Caixa; 2023. Available from: https://fundacionlacaixa.org/documents/d/guest/aprenentatge-servei-projectes-socioeducatius-pdf.

[59] Marques-Sule E, Chiva-Bartoll O, Carrasco JJ, Hernández-Guillén D, Pérez-Alenda S, Francisco-Garcés X et al. Impact of Service-Learning on Physiotherapy Students: Exercise Programs for Patients with Heart Transplantation and Acute Coronary Syndrome—A Randomized Clinical Trial. J Clin Med 2022;11(15):4360. 10.3390/jcm11154360. 10.3390/jcm11154360 PMC9369229 35955977

[60] Seif G, Coker-Bolt,Patty, Kraft, Sara, Gonsalves, Wanda, Simpson, Kit, and, Johnson E. The development of clinical reasoning and interprofessional behaviors: service-learning at a student-run free clinic. J Interprof Care. 2014;28(6):559–64. 10.3109/13561820.2014.921899. 10.3109/13561820.2014.921899 24865993

[61] Crawford E, Caine AM, Hunter L, Hill AE, Mandrusiak A, Anemaat L, et al. Service learning in developing countries: Student outcomes including personal successes, seeing the world in new ways, and developing as health professionals. J Interprofessional Educ Pract. 2017;9:74–81. 10.1016/j.xjep.2017.08.006.

[62] Marcilla-Toribio I, Moratalla-Cebrián ML, Bartolomé-Guitierrez R, Cebada-Sánchez S, Galán-Moya EM, Martínez-Andrés M. Impact of Service-Learning educational interventions on nursing students: An integrative review. Nurse Educ Today 2022;116:105417. 10.1016/j.nedt.2022.105417. 10.1016/j.nedt.2022.105417 35691112

[63] Zamora-Sánchez JJ, Zabaleta-del-Olmo E, Gea-Caballero V, Julián-Rochina I, Pérez-Tortajada G, Amblàs-Novellas J. Convergent and discriminative validity of the Frail-VIG index with the EQ-5D-3L in people cared for in primary health care. BMC Geriatr. 2021;21(1):243. 10.1186/s12877-021-02186-x. 33849481 10.1186/s12877-021-02186-x PMC8045391

[64] Zamora-Sánchez JJ, Zabaleta-del-Olmo E, Gea-Caballero V, Julián-Rochina I, Pérez-Tortajada G, Amblàs-Novellas J. Validez convergente y discriminativa del índice Frágil-VIG con la escala de Braden en personas atendidas en atención domiciliaria. Rev Esp Geriatría Gerontol. 2022;57(2):71–8. 10.1016/j.regg.2021.12.003. 10.1016/j.regg.2021.12.003 35307198

[65] León-Carrascosa V, Sánchez-Serrano S, Belando-Montoro MR. Diseño y validación de un cuestionario para evaluar la metodología Aprendizaje-Servicio. Estud Sobre Educ. 2020;39:247–66. 10.15581/004.39.247-266.

[66] World Medical Association. WMA Declaration of Helsinki: ethical principles for medical research involving human participants. Available from: https://www.wma.net/policies-post/wma-declaration-of-helsinki/. [cited 2025 Apr 23]. 10.1001/jama.2024.21972 39425955

