# Supplementary material for: A Service-Learning Program Based on Comprehensive Geriatric Assessment and Health Promotion in Older Adults: Protocol for the GEROS Project
Source: BMC Geriatr. 2026 Mar 21;26:602. doi: 10.1186/s12877-026-07312-1 (PMC13127011; doi:10.1186/s12877-026-07312-1)
Supplement: Supplementary file 1 — Supplementary Material 1. [file 12877_2026_7312_MOESM1_ESM.pdf]

SUPPLEMENTARY FILE 1: Questionnaires of the Study-Student and Older Adult

This supplementary file contains the instrument for assessing Service Learning by students, developed and validated by León Carrascosa et al. (1) as well as the two questionnaires specifically created for this study: one designed for students and another for older adult participants.

- Validated questionnaire Service-Learning (students)

Dimension	Item (English)	1= Very Little/none<br>5= Very much				
**Formative**	1. The service is related to the curricular content of my future profession.	1	2	3	4	5
	2. The service is linked to learning at university.	1	2	3	4	5
	3. The learning I have achieved is useful for my training as a professional.	1	2	3	4	5
	4. The learning I have achieved is useful for my personal development.	1	2	3	4	5
	5. I have gained practical knowledge through experience.	1	2	3	4	5
	6. I have developed the ability to learn in new contexts.	1	2	3	4	5
**Learning**	7. I consider that I have acquired greater responsibility for my professional performance.	1	2	3	4	5
	8. I have experienced personal during the service.	1	2	3	4	5
	9. I have developed the ability to organize and plan my time.	1	2	3	4	5
	10. The Service-Learning methodology has	1	2	3	4	5

	helped me to obtain tools tools for my professional future.					
	11. The experience has provided me with more social reflection linked to my future professional practice.	1	2	3	4	5
	12. I have shared reflections with different people on the practice of service-learning.	1	2	3	4	5
	13. Teamwork has made it easier for me to create knowledge networks.	1	2	3	4	5
	14. My communication skills have improved.	1	2	3	4	5
	15. I understand the meaning of service performed to help others.	1	2	3	4	5
	16. I have been aware of the need to link the reality of society with the university.	1	2	3	4	5
	17. The experience has increased my social sensivity.	1	2	3	4	5
**Service**	18. When necessary, I have made decisions for the smooth running of the service.	1	2	3	4	5
	19. I have taken initiative to put forward different points of view to organize the sessions.	1	2	3	4	5
	20. Overall, the service (project/program) has met my expectations.	1	2	3	4	5
	21. My service has responded to the needs of the institution/entity where the service is performed	1	2	3	4	5

	22. I have felt committed to the project.	1	2	3	4	5
	23. I have carried out activities according to the needs of the project participants.	1	2	3	4	5
	24. The distribution of tasks has been adequate.	1	2	3	4	5
	25. The project has been evaluated throughout its process.	1	2	3	4	5
	26. My participation has been recognised.	1	2	3	4	5
	27. I have participated in the organization and development of the project.	1	2	3	4	5
	28. I have participated in the coordination of activities during the service.	1	2	3	4	5
	29. My participation has satisfactorily responded to the needs of the institution/entity where the service is performed.	1	2	3	4	5

- **Questionnaire for students**

Rate from **0 to 5** the degree of impact the project has had on the following aspects (0 = not at all, 5 = very much).

1 = Not at all

2 = A little

3 = Fair

4 = Quite a lot

5 = Very much

- General satisfaction with the activity
- Knowledge about health promotion and disease prevention

- Skills and technical competencies regarding Comprehensive Geriatric Assessment
- Skills and technical competencies regarding Intrinsic Capacity (ICOPE)
- Broadening my interdisciplinary perspective
- Clinical reasoning ability
- Problem-solving ability
- Integration of the knowledge, skills, and competencies of the subject in which the activity was developed

Finally, do you have any comments or suggestions? (open question)

- **Questionnaire for older adults**

Rate from **0 to 5** the degree of impact the project has had on the following aspects (0 = no impact, 5 = very high impact).

1 = Not at all

2 = A little

3 = Fair

4 = Quite a lot

5 = Very much

1. General satisfaction with the activity
2. My knowledge about my own health has increased
3. My knowledge about health status and risk factors that I can modify to improve my health and prevent diseases has increased
4. My knowledge about available health resources in the community to improve my health has increased

Finally, do you have any comments or suggestions? *(open question)*

- **References**

1. León-Carrascosa V, Sánchez-Serrano S, Belando-Montoro MR. Diseño y validación de un cuestionario para evaluar la metodología Aprendizaje-Servicio. Estud Sobre Educ. 2020 Oct 1;39:247–66. doi: https://doi.org/10.15581/004.39.247-266
